# Multi-Hospital Pediatric Surge Response to an RSV Epidemic in a US State: Oregon, 2022

**DOI:** 10.1017/dmp.2025.10303

**Published:** 2026-02-05

**Authors:** Brendan Cleary, Matthew Hudkins, Peter Graven, Matthias Merkel, Carl Eriksson

**Affiliations:** 1 Pediatrics, University of Nebraska College of Medicine, Omaha, NE, USA; 2 Pediatrics, Oregon Health & Science University, Portland, OR, USA; 3 Department of Business Intelligence and Advanced Analytics, Oregon Health & Science University, Portland, OR, USA; 4 School of Public Health, Portland State University, Portland, OR, USA; 5 Department of Anesthesiology and Perioperative Medicine, Oregon Health & Science University, Portland, OR, USA

**Keywords:** Disaster Medicine, Disaster Planning, Surge Capacity, Vulnerable Populations, Hospital Bed Capacity

## Abstract

**Importance:**

There is limited data describing statewide pediatric surge response during times of capacity strain.

**Objective:**

Characterize the burden and response to a surge in pediatric respiratory admissions in Oregon in 2022.

**Design, Setting, and Participants:**

This analysis utilized data from the Oregon Capacity System (OCS) and the state discharge database to describe patient characteristics, census changes, and admission pattern shifts during an RSV epidemic in 2022.

**Main Outcome and Measure:**

Statewide pediatric census, weekly pediatric admissions, weekly admissions from non-children’s hospital emergency departments (EDs) to non-children’s hospitals.

**Results:**

The median census in Oregon’s pediatric inpatient hospitals increased by 19% during the surge period (306 vs 364, *P* < 0.001), while the median pediatric intensive care unit census increased by 50% (24 vs 36, *P* < 0.001). Weekly elective pediatric admissions to children’s hospitals decreased by 33% (30 vs 20, *P* = 0.03). ED admissions to non-children’s hospitals increased by 160% (15 vs 39 per week, *P* = 0.02).

**Conclusion and Relevance:**

As the statewide pediatric inpatient census increased, targeted reductions in elective admissions and increased utilization of non-children’s hospitals increased capacity during a respiratory surge. This analysis underscores the importance of real-time situational awareness and coordinated surge response between hospitals.

## Key Points

- **Question**: How did Oregon’s hospitals respond to a surge of children requiring hospitalization in 2022?
- **Findings:** In this retrospective analysis of statewide admission and capacity data, pediatric respiratory admissions across Oregon increased by 368% during the surge period. Median Pediatric Intensive Care Unit (PICU) census increased by 50%, and pediatric acute care census increased by 18%. Weekly elective admissions to children’s hospitals decreased by 33%.
- **Meaning**: Oregon’s inpatient pediatric census increased in all measured areas, highlighting a statewide collaborative effort in surge response. Limiting elective admissions created additional capacity for children.

## Introduction

The increased burden of disease related to Respiratory Syncytial Virus (RSV) in children during the 2022-2023 respiratory season in the United States strained hospitals and challenged statewide health systems.^1^^–^^3^ During the 2022-2023 respiratory season, national hospitalization rates for children with RSV exceeded 19 per 100,000, nearly twice the peak that had been seen in the previous half-decade and overshadowing the typical burden of nearly 80,000 annual RSV-related hospitalizations.^4^^–^^6^ Oregon experienced a 6-week period of more than 8 weekly admissions per 100,000 children, giving rise to a statewide disaster declaration.^7^ Leaders within Oregon’s public health agencies, children’s hospitals, and non-children’s hospitals collaborated to respond to this surge of acutely ill children.^8^

While effective disaster preparedness within hospitals is critical to saving lives during public health emergencies, there are limited quantitative studies describing responses to surge events.^9^^–^^11^ Capacity awareness is a vital aspect of crisis management, and the integration of bedspace monitoring systems is well described.^12^^,^^13^ In the last two decades, emergency response in pediatrics has been a focal point at a national level; individual states and municipalities have, in turn, begun capacity reporting and disaster management protocols.^6^^,^^14^^–^^16^ Despite this focus, large gaps in preparedness and service availability in times of crisis remain.^6^^,^^17^^–^^19^ Further, recent studies have highlighted the disconnect between national recommendations and empiric analyses of surge capacity.^20^

Pediatric hospital services in the United States have become more concentrated in children’s hospitals,^21^^,^^22^ which may decrease overall capacity for large pediatric surges. We analyzed statewide pediatric admission patterns and census in Oregon during the 2022 surge in RSV-related hospitalizations in order to better understand Oregon’s pediatric surge capacity and performance and add to the limited body of literature available on statewide pediatric surge capacity.^14^^,^^23^^,^^24^

## Methods

### Study Design

This study was a retrospective analysis of changes in statewide pediatric census and inpatient discharges during a 4-week surge period in November and December of 2022 compared to a pre-surge baseline period. The Oregon Health & Science University Institutional Review Board granted approval. Reporting of this study conforms to the STROBE statement.^25^

### Data

We analyzed data from the Oregon Capacity System (OCS) and Oregon discharge database (both available through Apprise Health Insights, Portland, OR).^26^^,^^27^ Beginning in the fall of 2022, the OCS tracks the hourly census for each unit in all 61 non-Veterans Affairs hospitals in Oregon using a real-time data feed from each hospital to capture admission, discharge, and transfer events. Pediatric units are divided into Neonatal Intensive Care Units (NICUs), Pediatric Intensive Care Units (PICUs), and non-ICU wards, which we refer to as “pediatric acute care” units. Two non-children’s hospitals’ data were excluded due to missing data during the study period. The discharge database details every Emergency Department (ED) and inpatient discharge from Oregon’s acute care hospitals starting in January 2019.

### Population

**Figure 1.**
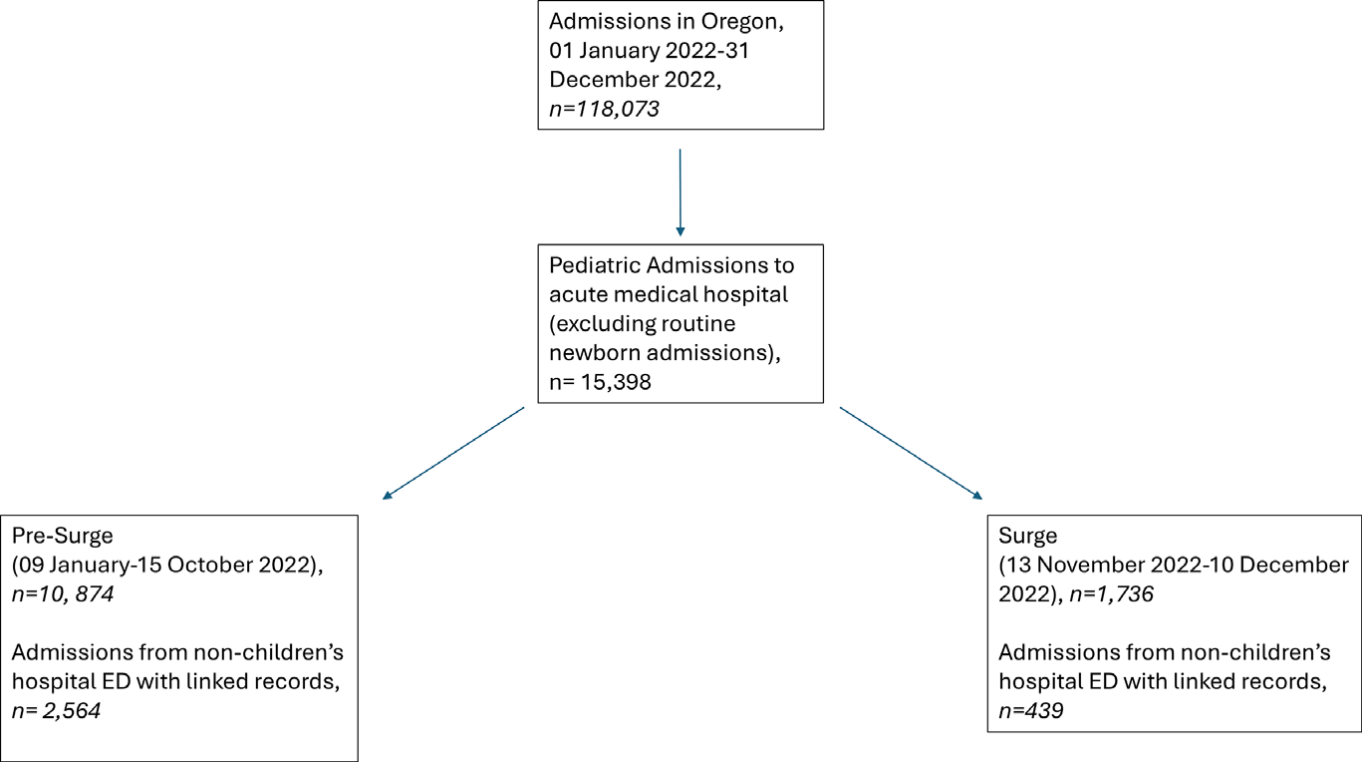
Flow diagram describing patient selection from Oregon discharge database.

For OCS analysis, we included all inpatient pediatric beds in every acute care medical hospital in Oregon with consistently available data from 18 September 2022 until 10 December 2022. We were unable to include earlier data as an updated OCS was implemented hospital-by-hospital in the fall of 2022. For analysis of discharge data (Figure 1), we included pediatric patients aged <18 years admitted from 09 January 2022 until 10 December 2022. We excluded admissions identified as normal newborns (Priority Type 04),^28^ as they utilize unique resources (e.g., post-partum units) that do not meaningfully contribute to pediatric acute bed infrastructure. Admissions to Oregon’s 2 inpatient pediatric psychiatric facilities were excluded, as these facilities do not provide acute inpatient medical care.

### Surge Period

We defined the surge period based on CDC-reported rates of RSV-related hospitalization for Oregon children.^5^ Hospitalizations peaked over a 6-week period (30 October through 10 December 2022), where weekly rates exceeded 8 per 100,000 children.^5^ We defined the surge period as the last 4 weeks of this peak (13 November through 10 December), during which we expected hospital census to be at its apex. We created a 4-week wash-in period (16 October through 12 November) during which RSV-related hospitalizations were rising. We defined our pre-surge period to reflect normal patterns of RSV admissions (less than 1 per 100,000 children as described in the RSV-Net database) in the preceding season.^5^ Our pre-surge period for OCS dataset analysis constituted a 4-week period (18 September-15 October); the pre-surge period for the discharge database started on 09 January, continuing through 15 October 2022.

### Discharge Categorization by Disease and Priority

In the discharge database, we categorized respiratory admissions using Major Diagnostic Category 04 (MDC-04). We also identified admissions with bronchiolitis (a clinical diagnosis often associated with RSV infection) using primary ICD-10 discharge diagnoses for bronchiolitis (J21.0, J21.1, J21.8, J21.9)^18^^,^^29^^–^^37^; we used this approach as variable testing for RSV may result in inaccurate documentation of RSV-related hospitalizations. We identified elective vs non-elective admissions using Medicare-defined priority code.^28^

### Outcome Measurement

The primary outcomes were median statewide pediatric census, measured hourly, by each type of unit (NICU, PICU, and pediatric acute care), and median statewide weekly admissions (Sunday through Saturday) during the surge period compared to the pre-surge period. To understand admission patterns from non-children’s hospital EDs, we also measured the median number of pediatric admissions from non-children’s hospital EDs who were admitted to a children’s hospital vs a non-children’s hospital.

### Statistical Analysis

For OCS data, available statewide census by unit type and total acute care pediatric census were calculated by summing the census on each individual unit. Median with interquartile ranges (IQR) for census were reported for each period and compared using the Wilcoxon rank-sum test.

For discharge data, categorical variables were presented as counts with percentages, and continuous variables as medians with IQR. Chi-square testing was used to compare categorical variables between pre-surge and surge periods, with the Wilcoxon rank-sum to compare weekly admissions by elective and diagnostic category for both the entire cohort and subdivided into children’s hospitals and non-children’s hospitals. To assess changes in admission patterns, we first categorized patient disposition using discharge codes from ED encounters. These were then associated with inpatient admission encounters using a de-identified patient linker and date matching. Duplicate records were discarded. Based on our assessment that children are unlikely to be transferred from a children’s hospital ED to an inpatient unit in a non-children’s hospital, we compared weekly admissions from non-children’s hospital EDs during the pre-surge and surge periods using the Wilcoxon rank-sum test, and the proportion admitted to children’s vs non-children’s hospitals using chi-square. Analysis was performed using R (version 4.2.2).^38^

## Results

Table 1 describes census changes in 23 included pediatric units. Compared with the pre-surge period, the median statewide census increased by 19% during the surge period (306 vs 364, *P* < 0.001). Census increased in all pediatric unit types; the largest increase was in PICU (24 vs 36, *P* < 0.001). Within the 4-week (672-hour) pre-surge period, there were 627 hourly observations, and 661 for the 4-week surge period.

**Table 1.** Census changes in pediatric units in Oregon from the Oregon Capacity System’s real-time census tracking before and during the 2022 RSV surge. Values are median hourly census (interquartile range, IQR), significance testing performed using Wilcoxon rank-sum test

	Pre-surge period (18 Sep-15 Oct 2022), median hourly census (IQR)	Surge period (13 Nov-10 Dec 2022), median hourly census (IQR)	Difference	*P* value
**Total pediatric census**	306 (295, 317)	364 (348, 383)	+58 (19%)	<0.001
**Acute care census**	137 (130, 145)	162 (153, 170)	+25 (18%)	<0.001
Children’s hospital (2 units)	113 (107, 120)	128 (120, 135)	+15 (13%)	<0.001
Non-children’s hospital (8 units)	24 (20, 27)	34 (29, 37)	+10 (42%)	<0.001
**PICU census**	24 (21, 26)	36 (33, 39)	+12 (50%)	<0.001
Children’s hospital (2 units)	24 (21, 26)	36 (33, 39)	+12 (50%)	<0.001
**NICU census**	147 (142, 150)	168 (156, 180)	+21 (14%)	<0.001
Children’s hospital (2 units)	71 (69, 79)	81 (79, 83)	+10 (14%)	<0.001
Non-children’s hospital (9 units)	72 (68, 76)	87 (76, 101)	+15 (21%)	<0.001

Table 2 describes the demographic characteristics of pediatric admissions during the pre-surge (10,874 admissions) and surge (1,736 admissions) periods.

**Table 2.** Demographic characteristics of pediatric admissions to Oregon hospitals before and during the 2022 RSV surge. Categorical variables (gender, race, ethnicity, primary insurance) were compared using chi-squared analysis, and Wilcoxon rank-sum was used for continuous variables (age)

	Pre-surge period (09 Jan-15 Oct 2022)	Surge period (13 Nov-10 Dec 2022)	*P* value
**Total number of admissions**	10,874	1,736	
**Median age (interquartile range)**	5 (0, 13)	2 (0, 7)	<0.001
**Gender (%)**			0.06
Female	5,115 (47)	772 (44)	
Male	5,759 (53)	961 (55)	
Undetermined	0 (0)	3 (0.2)	
**Race (%)**			<0.001
Asian	386 (3.5)	33 (1.9)	
Black/African American	478 (4.4)	63 (3.6)	
White	7,122 (65)	1,144 (66)	
Other^a^	1,818 (17)	287 (17)	
Unknown/declined to answer	1,070 (10)	209 (12)	
**Ethnicity (%)**			0.04
Hispanic or Latino	2,029 (19)	312 (18)	
Non-Hispanic or Latino	7,779 (72)	1,220 (70)	
Unknown/declined to answer	1,066 (10)	204 (12)	
**Primary insurance (%)**			0.8
Public	6,175 (57)	987 (57)	
Private	4,266 (39)	675 (39)	
Other or unknown	433 (4.0)	74 (4.3)	

*Note.* ^a^ Other category includes American Indian/Native Alaskan, Native Hawaiian/Pacific Islander, or self-identified “Other.”

**Figure 2.**
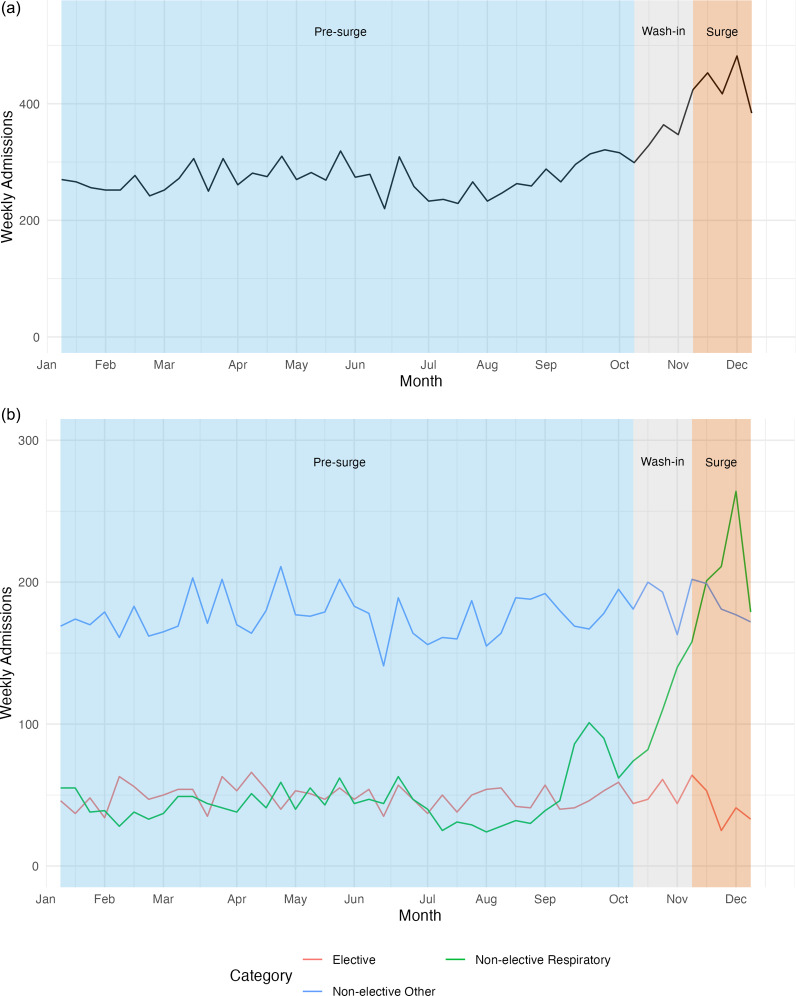
Pediatric inpatient admissions in Oregon, 2022. (a) Total pediatric admissions and (b) Elective, non-elective respiratory (MDC 04), and non-elective other (non-respiratory) admissions. Non-elective respiratory admissions increased during the surge period while statewide elective admissions decreased.

Total weekly pediatric admissions increased from 270 during the pre-surge period to 435 during the surge (*P* < 0.001, Figure 2 and Table 3). Non-elective respiratory admissions increased from 44 to 206 per week (*P* < 0.001). Categorizing by ICD-10-based discharge diagnosis, non-elective bronchiolitis admissions increased from 9 to 102 (*P* < 0.001) during the surge period. The median weekly elective admissions decreased from 50 during pre-surge to 37 during the surge period (*P* = 0.06), with a significant decrease in weekly elective admissions at children’s hospitals (30-20, *P* = 0.03).

**Table 3.** Weekly admissions to Oregon’s children’s and non-children’s hospitals by admission priority, type, and diagnosis. Values are median weekly admissions with interquartile ranges (IQR). Significance testing was performed using Wilcoxon rank-sum test

	Pre-surge period (09 Jan-15 Oct 2022) median (IQR)	Surge period (13 Nov-10 Dec 2022) median (IQR)	Difference	*P* value
**State of Oregon** (n = total pediatric admissions)	n = 10,874 over 40 weeks	n = 1,736 over 4 weeks		
Total	270 (252, 292)	435 (401, 468)	+165 (61%)	<0.001
Elective	50 (42, 54)	37 (29, 47)	−13 (26%)	0.06
Non-elective respiratory	44 (38, 55)	206 (190, 238)	+162 (368%)	<0.001
Non-elective non-respiratory	177 (165, 185)	179 (175, 190)	+2 (1%)	0.4
Bronchiolitis	9 (6, 12)	102 (88, 121)	+93 (1,030%)	<0.001
**Children’s hospitals** (n = total pediatric admissions)	n = 7,054 over 40 weeks	n = 984 over 4 weeks		
Total	176 (167, 184)	246 (226, 267)	+70 (40%)	<0.001
Elective	30 (26, 35)	20 (15, 27)	−10 (33%)	0.03
Non-elective respiratory	27 (23, 33)	118 (106, 124)	+91 (337%)	<0.001
Non-elective non-respiratory	116 (109, 121)	108 (103, 118)	−8 (7%)	0.3
Bronchiolitis	5 (3, 7)	49 (43, 54)	+44 (880%)	<0.001
**Non-children’s hospitals** (n = total pediatric admissions)	n = 3,820 over 40 weeks	n = 752 over 4 weeks		
Total	96 (85, 108)	178 (173, 204)	+82 (85%)	<0.001
Elective	18 (16, 21)	17 (15, 20)	−1 (5%)	0.6
Non-elective respiratory	17 (11, 21)	90 (83, 116)	+73 (429%)	<0.001
Non-elective non-respiratory	59 (56, 64)	72 (71, 73)	+13 (22%)	0.05
Bronchiolitis	4 (2, 5)	52 (45, 67)	+48 (1,200%)	<0.001

**Figure 3.**
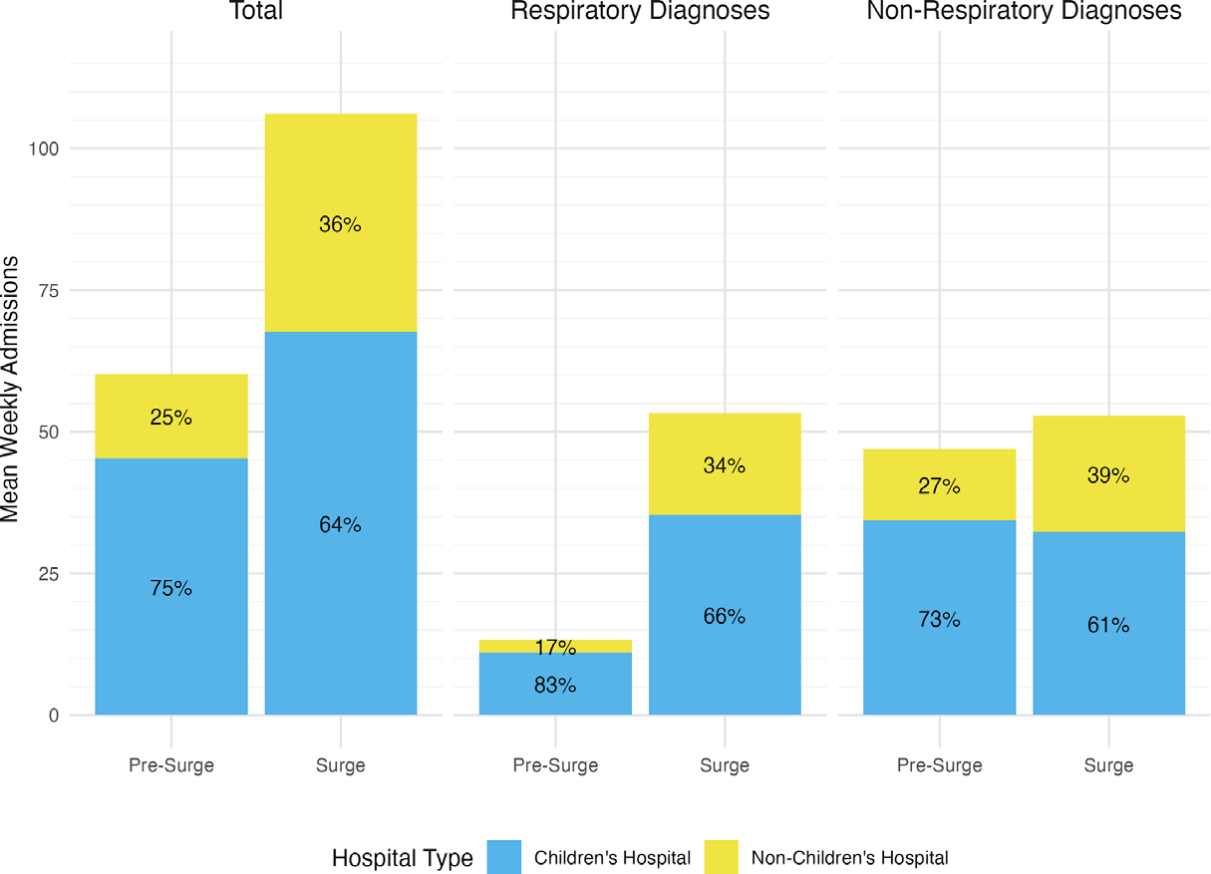
Admissions from non-children’s hospital Emergency Departments to children’s vs non-children’s hospitals before and during the 2022 RSV surge. With the exception of non-respiratory admissions to children’s hospitals, all changes in percentages of patients admitted to children’s vs non-children’s hospitals are statistically significant using chi-square testing (*P* < 0.001).

After discarding duplicate records, we identified 2,829 admissions from non-children’s hospital EDs. Weekly admissions from non-children’s hospital EDs increased by 66% during the surge period (59 vs 98, *P* = 0.03, Table 4). Among admissions from non-children’s hospitals, the percentage of patients admitted to a non-children’s hospital increased from 25% to 36% (*P* < 0.001), with increases both in patients with respiratory diagnoses (17% admitted to non-children’s hospital pre-surge and 34% admitted to non-children’s hospital during surge, *P* < 0.001, Figure 3) and patients with non-respiratory diagnoses (27% admitted to non-children’s hospitals pre-surge and 39% admitted to non-children’s hospitals during surge, *P* < 0.001).

**Table 4.** Admissions from non-children’s hospital Emergency Departments and inpatient units to children’s hospitals before and during the 2022 RSV surge. The values are median weekly admissions. Significance testing was performed using Wilcoxon rank-sum test

	Pre-surge period (18 Sep-15 Oct 2022) (n = 2,564)	Surge period (13 Nov-10 Dec 2022) (n = 439)	Difference (percent change)	*P* value
**Statewide admissions from non-children’s hospital Emergency Departments, per week**	59	98	+39 (66%)	0.03
**Children’s hospital admissions per week** (proportion of total admissions)	45	60	+15 (33%)	0.05
Respiratory	10	30	+20 (200%)	0.03
Non-respiratory	34	29	−5 (15%)	0.2
**Non-children’s hospital admissions per week** (proportion of total admissions)	15	39	+24 (160%)	0.02
Respiratory	3	17	+14 (467%)	0.006
Non-respiratory	12	21	+9 (75%)	0.3

## Discussion

Using a novel data source based on real-time automatic feeds from Oregon’s acute care hospitals, we identified a 50% increase in statewide PICU census during a 4-week period of increased RSV-related admissions in 2022, and an almost 20% increase in statewide pediatric census overall. While weekly pediatric admissions increased by 61%, we also identified a 33% reduction in elective admissions among Oregon’s children’s hospitals during the surge period compared to pre-surge. Among children presenting to a non-children’s hospital ED who needed admission, we found a significant increase in the percentage of children admitted to a non-children’s hospital. These findings demonstrate a significant response to the surge across Oregon’s children’s and non-children’s hospitals.

In this study, statewide pediatric census increased across all units but was most pronounced in the PICU. Respiratory disease in children can require care only available in the PICU (such as positive-pressure ventilation and advanced airway management); meanwhile, the labor-intensive, highly specialized, and expensive nature of pediatric critical care limits staffing and bed space. As inpatient pediatric care is becoming increasingly concentrated in large dedicated pediatric centers, these facilities are exposed to a greater risk of overwhelm in times of high demand.^21^ The centralization of PICU resources in children’s hospitals may improve efficiency during normal operations, but reliance on a smaller number of hospitals can constrain service delivery and create capacity vulnerabilities in periods of high occupancy.^21^^,^^39^ There has been concern that regionalization challenges the delivery of timely and effective care, particularly for patients originating from rural and geographically isolated locations.^6^^,^^40^^–^^42^. In addition, there is evidence of significant pediatric load imbalance between hospitals during the 2022 viral respiratory season.^43^ Our analysis highlights the potential impact of load balancing during a surge in a highly centralized system.^15^^,^^21^^,^^44^

This analysis illustrates the important surge response role of different types of inpatient units in both children’s hospitals and non-children’s hospitals. PICU census increased significantly, as did census in acute care units in children’s hospitals. Importantly, non-children’s hospitals experienced an increase in median census by 42%, while weekly admissions from non-children’s hospital EDs increased by 66%; NICUs in both children’s and non-children’s hospitals saw similarly increased admissions and census. In order to accomplish this, non-children’s hospitals cared for an increased proportion of appropriately triaged ill infants and children that would have otherwise overwhelmed the dedicated children’s hospitals. Notably, our statewide coordination was primarily focused on hospitals with dedicated pediatric hospitalist or NICU teams; it is possible that widening this approach to include additional non-children’s hospitals could result in a more effective statewide surge response. This strategy may be most successful if it focuses on older, less critically ill children, and if it includes access to pediatric expertise (likely utilizing local pediatricians or remote experts through telemedicine).

While curtailing elective admissions is a recommended strategy to liberate scarce resources during a surge of admissions,^45^ there is little empiric data describing real-world applications of this strategy outside of extreme limitations on elective admissions during the early COVID-19 pandemic.^7^^,^^46^^–^^48^ We identified a 33% reduction in pediatric elective admissions to children’s hospitals during the RSV surge. By reducing elective admissions, we were able to free multiple critical resources: bedspace, staff, equipment, and supplies. While the overall number of decreased elective admissions was much lower than the increased bronchiolitis admissions, freeing even a small number of beds may have had a dramatic impact on additional children who had access to potentially life-saving care. Balancing the needs of patients directly affected by RSV with the less time-sensitive but often no less important needs of patients with scheduled admissions is difficult. As an example, our hospital only curtailed admissions for patients whose care could be delayed by 6 weeks without adverse health consequences, while continuing to admit patients for scheduled chemotherapy and other more urgent needs that were deemed inappropriate to delay. Importantly, delaying elective admissions resulted in an increased number of patients needing these admissions after the RSV surge, requiring additional planning and resources to meet these patients’ needs over a roughly 3-month period after the surge.

Among children who presented to non-children’s hospital EDs and required admission, we identified a significant increase in admissions to non-children’s hospitals, indicating an admission pattern shift that more deftly utilized these resources. Prior studies have described decreases in patient transfers during times of capacity strain.^48^ While our analysis revealed an increase in admissions to children’s hospitals from non-children’s hospitals, the net effect of load balancing operations was an increase in utilization of pediatric bedspace in non-children’s hospitals. These changes in admission patterns during the surge highlight our statewide cooperative effort to triage and treat an overwhelming number of ill infants and children. While both children’s hospitals and non-children’s hospitals experienced increases in census and admissions, non-children’s hospitals in Oregon absorbed a proportionally larger population of both respiratory and non-respiratory admissions during the Surge. This pattern illustrates a collaborative effort to load balance to meet the overwhelming pediatric service demand. The effect cannot be overstated: statewide partners substantially ameliorated the stress on dedicated children’s hospitals during this crisis.

In addition to utilizing statewide discharge data, our study utilized a novel data source that tracks unit-level census in real time. In the past, gathering this information within multiple hospital systems has often required manual input, which can be time-consuming and may not provide the same level of accuracy and granularity.^13^ Using this novel data source allowed us to understand how census changed on different types of pediatric units, highlighting the contributions of acute care units, NICUs, and PICUs in the surge response. Similar data sources could be valuable in understanding surge response in the future at sub-state, state, and multistate levels. Characterizing operational changes in pediatric care during the RSV Surge required review of data from 61 hospitals spread across 98,000 square miles of geographically and culturally diverse territory.

## Limitations

There were limitations to this study. While system behavior was retrospectively observed through data analysis, protocols and policies for individual hospitals relative to resource allocation were not reviewed. Notwithstanding the Governor’s Emergency Declaration^7^^,^^49^ and the Oregon Medical Coordination Center (OMCC), there were no centralized command and control agencies to direct independent medical facilities in their allocation of bedspace. The OMCC matched patients needing care to hospitals with the capability and capacity to provide this care when normal transfer mechanisms had failed,^50^ augmenting efforts from pediatric experts at our children’s hospital, who often historically aided in identifying alternate resources if their facility was unable to accommodate a patient. As OMCC’s scope does not include mandating admission of specific patients or adoption of surge principles, the response characterized in our study may reflect disparate adoption of guidance from regional pediatric agencies.^51^^,^^52^

Our study was limited to acutely ill children ages 0 days to 18 years. Adult units were not scrutinized, and as such, the census impacts of adolescent admissions to those beds were not assessed. However, because these admissions are infrequent, the effect is thought to be small. The care of adolescent patients in adult units, when appropriate, is useful in decompressing pediatric bed space demand but was not universally adopted in our state. Future studies to explore patient placement alternatives in disaster management are warranted.

Our discharge dataset contains elements that are based on administrative coding (e.g., primary discharge diagnosis, admission priority), which may be inaccurate. Determining the “elective” nature of an admission may be accurate based on source codes and diagnosis, but “elective” admissions represent a spectrum of urgency and include scheduled admissions that many would consider non-elective (e.g., admission for chemotherapy to treat cancer).

Finally, while RSV was a major driver of pediatric admissions during the surge period, increased rates of influenza, human metapneumovirus, and related enterovirus infections were also seen.^51^ Recognizing that no administrative data can perfectly capture the burden of a respiratory viral surge, we selected a broad MCD code for respiratory illness and ICD-10-based diagnostic codes reflecting admission for bronchiolitis. Our findings reflect an aggregate pediatric respiratory surge that, while predominantly driven by RSV, may also include other causative agents. The cumulative effect of the burden of respiratory disease was encapsulated within our study design but imperfectly categorizes instances of multiple pathology (such as RSV bronchiolitis *and* a bacterial superinfection). In our real-world experience, these cases were the rare exception to the typical respiratory admission.

## Conclusion

In response to a surge of patients requiring admission for RSV in 2022, hospital systems in Oregon increased PICU census by 50% and pediatric acute care census by 18% over a 4-week period. As respiratory admissions increased significantly, children’s hospitals reduced elective admissions by 33% to accommodate the increased demand. This study highlights the value of real-time statewide census monitoring systems in understanding pediatric inpatient resource utilization during a public health emergency, as well as the need for robust, collaborative pediatric disaster planning.
